# Efficacy of the hybrid closed-loop insulin delivery system in children and adolescents with type 1 diabetes: a meta-analysis with trial sequential analysis

**DOI:** 10.20945/2359-4292-2023-0280

**Published:** 2024-04-11

**Authors:** Rafael Oliva Morgado Ferreira, Talita Trevisan, Eric Pasqualotto, Pedro Schmidt, Matheus Pedrotti Chavez, Janine Midori Figueiredo Watanabe, Simone van de Sande-Lee

**Affiliations:** 1 Universidade Federal de Santa Catarina Florianópolis SC Brasil Universidade Federal de Santa Catarina, Florianópolis, SC, Brasil; 2 Clínica particular, Talita Trevisan Endocrinologia Itajaí SC Brasil Clínica particular, Talita Trevisan Endocrinologia, Itajaí, SC, Brasil; 3 Universidade Estadual do Piauí Teresina PI Brasil Universidade Estadual do Piauí, Teresina, PI, Brasil

**Keywords:** Closed-loop, glucose control, diabetes technology, type 1 diabetes, meta-analysis

## Abstract

The aim of this study was to assess the efficacy and safety of hybrid closed-loop (HCL) systems for insulin delivery in children and adolescents with type 1 diabetes (T1D). We searched Embase, PubMed, and Cochrane Library for randomized controlled trials (RCTs) published until March 2023 comparing the HCL therapy with control therapies for children and adolescents with T1D. We computed weighted mean differences (WMDs) for continuous outcomes and risk ratios (RRs) with 95% confidence intervals (CIs) for binary endpoints. Four RCTs and 501 patients were included, of whom 323 were randomized to HCL therapy. Compared with control therapies, HCL significantly improved the period during which glucose level was 70-180 mg/dL (WMD 10.89%, 95% CI 8.22-13.56%) and the number of participants with glycated hemoglobin (HbA1c) level < 7% (RR 2.61, 95% CI 1.29-5.28). Also, HCL significantly reduced the time during which glucose level was > 180 mg/dL (WMD -10.46%, 95% CI -13.99 to -6.93%) and the mean levels of glucose (WMD -16.67 mg/dL, 95% CI -22.25 to -11.09 mg/dL) and HbA1c (WMD -0.50%, 95% CI -0.68 to -0.31). There were no significant differences between therapies regarding time during which glucose level was < 70 mg/dL or <54 mg/dL or number of episodes of ketoacidosis, hyperglycemia, and hypoglycemia. In this meta-analysis, HCL compared with control therapies was associated with improved time in range and HbA1c control in children and adolescents with T1D and a similar profile of side effects. These findings support the efficacy of HCL in the treatment of T1D in this population.

## INTRODUCTION

Type 1 diabetes (T1D) is a complex, heterogeneous, and demanding condition that affects about 8.4 million people worldwide (1–4). Challenges in T1D management are greater in youths, primarily due to their dependence on parents and other caregivers like relatives and teachers. Other important difficulties include the high variation in insulin requirement and insulin sensitivity, potential erratic food intake (mainly in toddlers), and unpredictable activity patterns with a potential increased risk of episodes of hypoglycemia, which are difficult for children to communicate (5).

In this regard, hybrid closed-loop (HCL) systems have emerged as a promising alternative for T1D treatment, sustaining better glycemic results and improving the quality of life of the patients and their caregivers (6,7). The HCL system utilizes automated basal insulin delivery through an insulin pump integrated with continuous glucose monitoring (CGM) and an algorithm. It still requires the user (or caregiver) to manually administer the insulin bolus dose at mealtimes. In randomized controlled trials (RCTs) involving different systems and participants aged 7 years and older, HCL has been associated with an increased time in range (TIR) without increased risk of hypoglycemia when compared with sensor-augmented pump, low-glucose suspend, or predictive low-glucose suspend systems (6,8–17).

Previous systematic reviews and meta-analyses including both adults and children have shown efficacy and safety of HCL over control therapies (18–20). However, these studies presented combined outcomes from children and adults, since analyses specifically focused on children were unfeasible (21,22). Thus, the aim of this study was to perform a systematic review and meta-analysis of RCTs to evaluate the efficacy and safety of HCL treatment in children and adolescents with T1D.

## MATERIAL AND METHODS

The protocol of this systematic review and meta-analysis was registered in the International Prospective Register of Systematic Reviews (PROSPERO) under the registration number CRD42023412405.

### Eligibility criteria

Inclusion in this meta-analysis was restricted to studies that met the following eligibility criteria: (A) randomized controlled design, (B) comparison of HCL *versus* control therapies, (C) enrollment of children and adolescents (2 to 18 years) with T1D, (D) minimum follow-up of 4 weeks, (E) reporting of any outcome of interest, and (F) inclusion of patients treated with insulin for at least 6 months before enrollment.

### Search strategy and data extraction

We systematically searched PubMed, Embase, and Cochrane Library from inception to March 2023 using the following research strategy: ("closed-loop" OR "artificial pancreas" OR "automated insulin delivery" OR "artificial beta cell") AND (diabetes) AND (children OR infants OR adolescents). The references from all included studies, previous systematic reviews, and meta-analyses were also searched manually for any additional studies. Two authors (R.F and P.H.S) independently extracted the data following predefined search criteria and quality assessment.

### Endpoints and subgroup analyses

The efficacy outcomes included the percentage of time during which glucose level (A) remained within the target range (TIR; 70-180 mg/dL) during 24 hours, the percentage of time during which glucose level was (B) < 70 mg/dL, (C) < 54 mg/dL, and (D) > 180 mg/dL, (E) the mean glycated hemoglobin (HbA1c) level, (F) the number of patients with mean HbA1c < 7%, (G) the mean glucose level, and the number of events of (H) hyperglycemia, (I) ketoacidosis, and (J) severe hypoglycemia.

*Post hoc* subgroup analyses included data restricted to patients who (A) received intervention with the HCL system t:slim X2 insulin pump with Control-IQ Technology, (B) underwent control therapy with CGM, and (C) had at least 12 months since the T1D diagnosis.

### Quality assessment

The risk of bias assessment followed the recommendations of the Cochrane Handbook for Systematic Reviews of Interventions, with the Cochrane Collaboration’s tool for assessing the risk of bias in randomized trials (RoB 2) (23). Two authors (M.P.C. and E.P.) independently assessed the risk of bias, and disagreements were resolved with the senior author (S.S.L). Potential publication bias was evaluated using visual inspection of funnel plots (24). We also assessed the effects of influential studies on the pooled results by sequentially removing one study’s data and reanalyzing the remaining data (leave-one-out analysis) to ensure the stability of the pooled analysis effect (25).

The overall quality of evidence was assessed using the Grading of Recommendation, Assessment, Development, and Evaluations (GRADE) guidelines (26). The quality of evidence of the studies was rated as very low, low, moderate, or high based on the presence of risk of bias, inconsistency of results, imprecision, publication bias, and magnitude of treatment effects (27).

### Statistical analysis

This systematic review and meta-analysis followed the Cochrane recommendations and the Preferred Reporting Items for Systematic Review and Meta-Analyses (PRISMA) statement (28). Risk ratios (RRs) with 95% confidence intervals (CIs) were used to compare binary endpoints, and weighted mean differences (WMDs) were applied to compare continuous outcomes. Heterogeneity was assessed using Cochran’s Q test and I2 statistics; p values < 0.10 and I2 values ≥ 25% were considered significant (29). We used a fixed-effect model for endpoints considered to have low heterogeneity. DerSimonian and Laird random-effects models were used in outcomes with significant heterogeneity (30). The Cochrane Handbook for Systematic Reviews of Interventions was used for data conversion (31). Statistical analyses were performed using the software R, version 4.2.2 (R Core Team, 2021, Vienna, Austria).

### Trial sequential analysis

We performed a trial sequential analysis (TSA) of the studies included in the present meta-analysis to evaluate the reliability and conclusiveness of the available evidence. Our statistical plan included two-sided testing with a type I error of 5% and a statistical power of 80%. Both conventional and trial sequential monitoring boundaries for HCL and control groups were generated. The heterogeneity correction in the TSA was set to variance-based, and a fixed-effects model was applied. A z-score curve was generated to evaluate the confidence and adequacy of the evidence. An analysis was also performed to calculate the number of patients required in a meta-analysis to accept or reject the intervention. For statistical analysis, we used the software Trial Sequential Analysis, version 0.9 (Copenhagen Trial Unit, Centre for Clinical Intervention Research, Rigshospitalet, Copenhagen, Denmark) (32).

## RESULTS

### Study selection and characteristics

**Figure 1:**
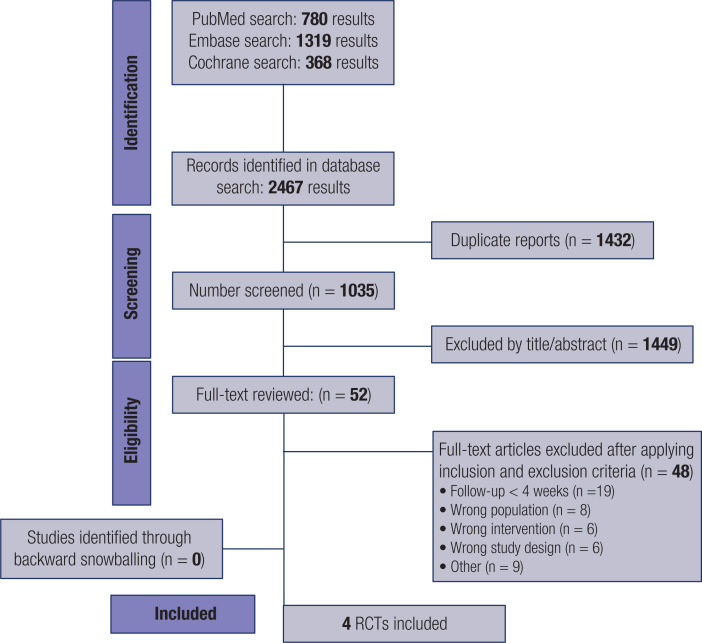
PRISMA flow diagram of study screening and selection. The search strategy in Embase, PubMed, and Cochrane yielded 2467 studies, of which 52 were thoroughly reviewed for inclusion and exclusion criteria. A total of four studies were included in the meta-analysis.

As illustrated in Figure 1, the search yielded 2,467 results. After removing duplicates and ineligible studies by title or abstract, we performed a full-text review of 52 studies. Of these, four were included in the present meta-analysis (5,9,33,34). The main reasons for exclusion were follow-up periods shorter than 4 weeks, inclusion of nontarget populations, and studies without a randomized controlled design (*e.g.*, crossover or single-arm design). A total of 501 patients were included, of whom 323 (64.4%) were treated with HCL therapy. The mean age ranged from 3.84 to 13.1 years, and the follow-up duration ranged from 13 weeks to 6 months. Most baseline characteristics were comparable between groups, as shown in Table 1.

**Table 1:** Characteristics of the studies included in the present meta-analysis

Study	Number of patients, n (%)		Age (years), mean (SD)		Male to Female ratio, n		Type of HCL	Control therapy	Time since diagnosis	HbA1c (%), mean (SD)		Follow-up
	HCL	CT	HCL	CT	HCL	CT				HCL	CT	
Breton and cols. (2020)	78 (77.2)	23 (22.8)	11.3 (2)	10.8 (2.4)	40:38	11:12	t:slim X2 insulin pump with Control-IQ Technology plus an algorithm developed at the University of Virginia	Insulin pump plus CGM	At least 12 months	7.6 (1)	7.4 (0.6)	16 weeks
Messer and cols. (2022)	112 (67.9)	53 (33.1)	12 (3)	12 (3)	57:55	38:15	iLet pump with the Cambridge closed-loop algorithm	Insulin pump or multiple daily injections plus CGM	At least 12 months	8.1 (1.2)	7.8 (1.1)	13 weeks
Wadwa and cols. (2023)	68 (66.7)	34 (33.3)	3.84 (1.23)	4.06 (1.25)	35:33	15:19	t:slim X2 insulin pump with Control-IQ Technology	Insulin pump or multiple daily injections plus CGM	At least 6 months	7.5 (1.2)	7.7 (0.9)	13 weeks
Ware and cols. (2022)	65 (48.9)	68 (51.1)	13.1 (2.6)	12.8 (2.9)	37:28	39:29	Insulin pump that ran the Cambridge proprietary model predictive control algorithm	Insulin pump with or without CGM	At least 12 months	8.2 (0.7)	8.3 (0.8)	24 months

Abbreviations: CGM, continuous glucose monitoring; CT, control therapy; HbA1c, glycated hemoglobin; HCL, hybrid closed-loop; SD, standard deviation.

### Pooled analysis of all studies

**Figure 2:**
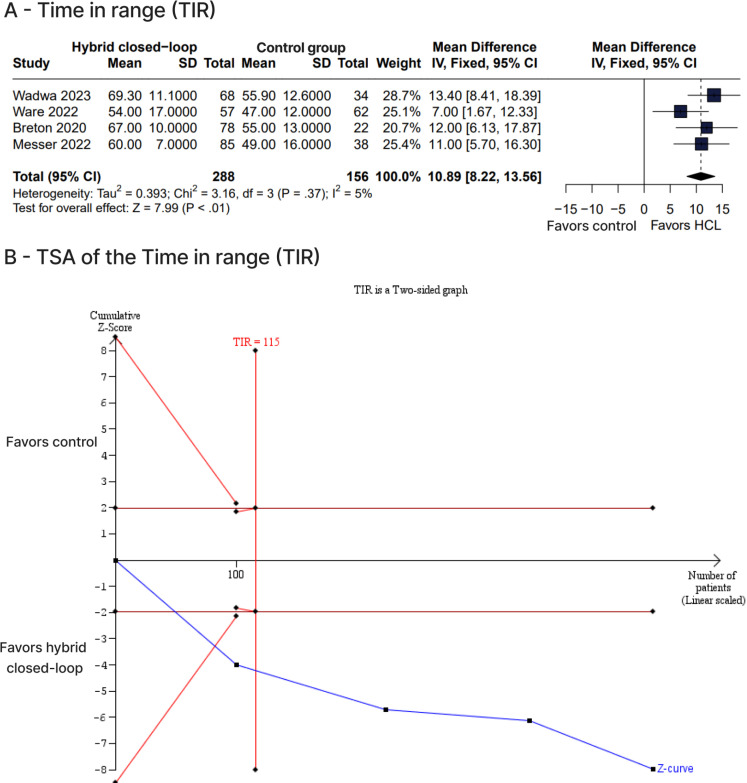
**(A)** Forest plot comparing hybrid closed-loop versus control therapies for the time in range; **(B)** Trial sequential analysis of four studies for the time in range.

**Figure 3:**
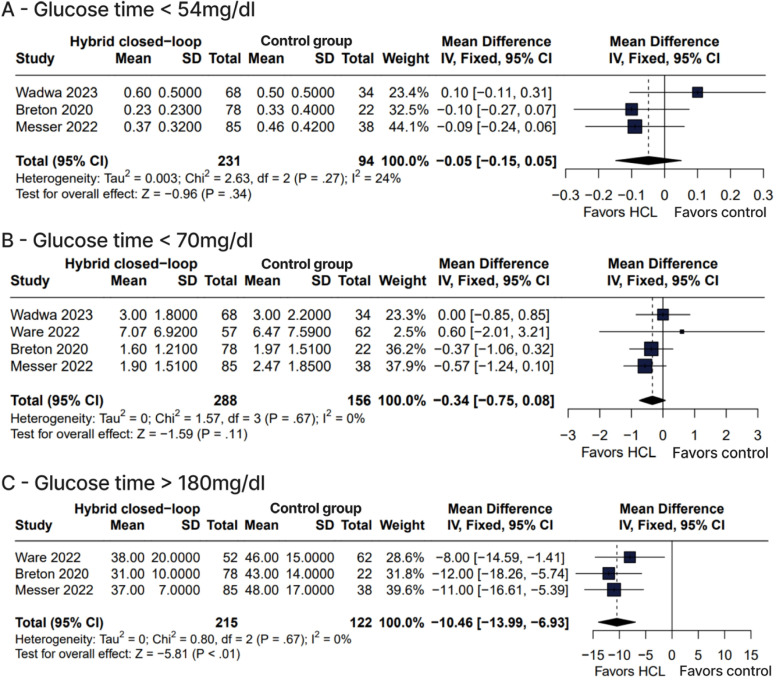
Glucose time <54 mg/dl (A) and glucose time <70 mg/dl (B) were not significantly different between groups. Glucose time >180 mg/dl (C) was significantly lower in the hybrid closed-loop group.

**Figure S1:**
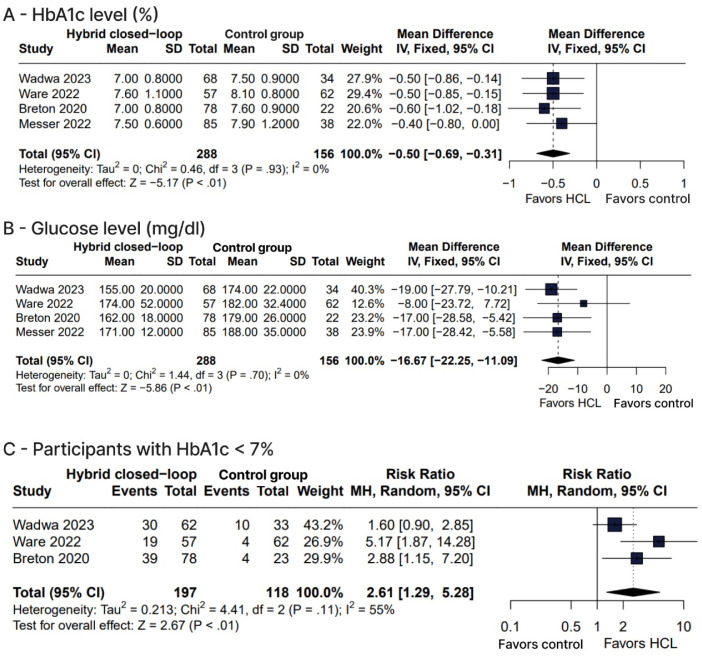
Glycated hemoglobin (HbA1c) level (**A**) and glucose level (**B**) were significantly lower in the hybrid closed-loop group. The number of patients with HbA1c <7% (**C**) was significantly greater in the hybrid closed-loop group.

**Figure S2:**
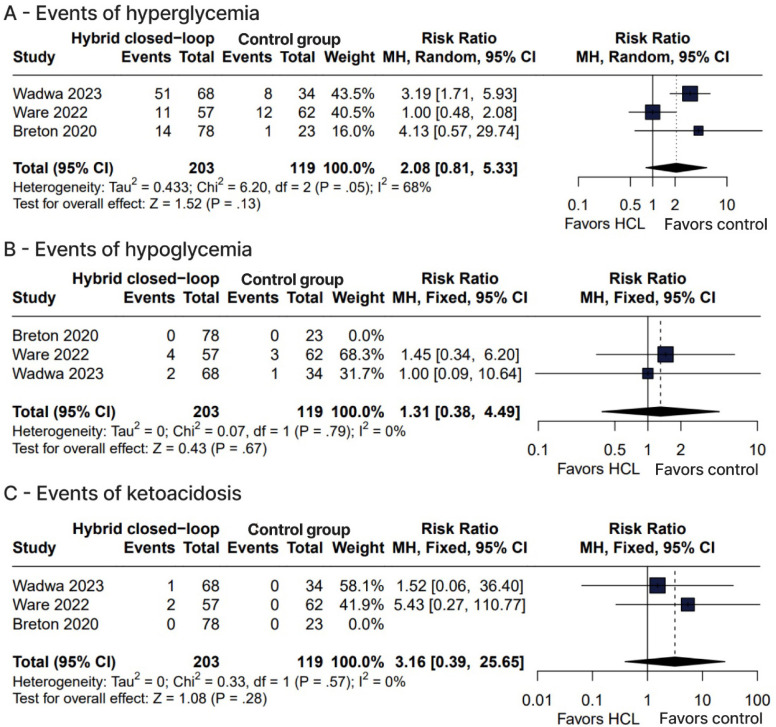
Events of hyperglycemia (**A**), hypoglycemia (**B**), and ketoacidosis (**C**) were not significantly different between groups.

The percentage of time during which glucose level was in the target range of 70-180 mg/dL was significantly greater with HCL compared with control therapies (WMD 10.89%, 95% CI 8.22-13.56%, p < 0.01, I^2^ = 5%) (Figure 2A). Similarly, the number of patients who reached a mean HbA1c level < 7% was greater with HCL *versus* control therapies (RR 2.61, 95% CI 1.29-5.28, p < 0.01, I^2^ = 55%) (Supplementary Figure S1C). The HCL therapy compared with control therapies also had a lower percentage of time during which glucose level was > 180 mg/dL (WMD -10.46%, 95% CI -13.99 to -6.93%, p < 0.01, I^2^ = 0%) (Figure 3C) and lower mean levels of glucose (WMD -16.67 mg/dL, 95% CI -22.25 to -11.09 mg/dL, p < 0.01, I^2^=0%) (Supplementary Figure S1B) and HbA1c (WMD -0.50%, 95% CI -0.68 to -0.31%, p < 0.01, I^2^ = 0%) (Supplementary Figure S1A). No significant differences between therapy groups were observed regarding percentage of time during which glucose level was < 70 mg/dL (WMD -0.34 mg/dL, 95% CI -0.75-0.08 mg/dL, p = 0.11, I^2^ = 0%) (Figure 3B) and < 54 mg/dL (WMD -0.05 mg/dL, 95% CI -0.15-0.05 mg/dL, p = 0.27, I^2^ = 24%) (Figure 3A). In terms of adverse events, the number of events of ketoacidosis (RR 3.16, 95% CI 0.39-25.64, p = 0.57, I^2^ = 0%) (Supplementary Figure S2C), hyperglycemia (RR 2.08, 95% CI 0.81-5.33, p = 0.13, I^2^ = 68%) (Supplementary Figure S2A), and hypoglycemia (RR 1.31, 95% CI 0.38-4.49, p = 0.67, I^2^ = 0%) (Supplementary Figure S2B) were not significantly different between groups.

### Subgroup analyses

**Figure S3:**
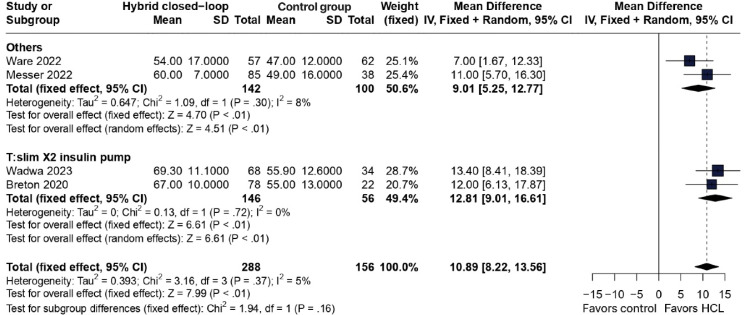
Forest plot of the time in range (TIR) for the t:slim X2 insulin pump with Control-IQ Technology.

In the subgroup analysis of the HCL system t:slim X2 insulin pump, the TIR (WMD 12.81%, 95% CI 9.01-16.61%, p < 0.01, I^2^ = 0%) (Supplementary Figure S3) and number of patients who reached HbA1c level < 7% (RR 1.93, 95% CI 1.11-3.34, p = 0.02, I^2^ = 16%) were greater with HCL than control treatments. The HCL group also had greater reductions in glucose level (WMD -18.27 mg/dL, 95% CI -25.27 to -11.27 mg/dL, p < 0.01, I^2^ = 0%) and HbA1c level (WMD -0.54%, 95% CI -0.81 to -0.27%, p < 0.01, I^2^ = 0%) compared with the control group.

**Figure S4:**
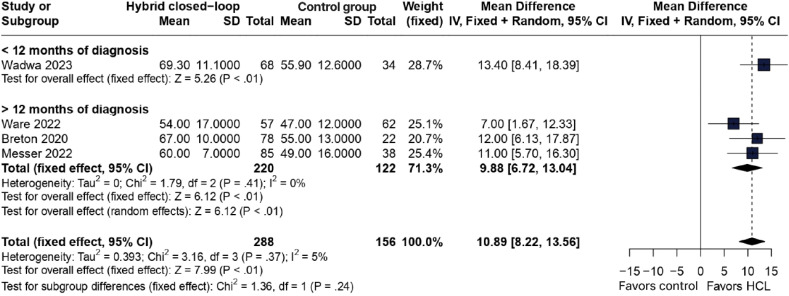
Forest plot of the time in range (TIR) analyzing the time since diagnosis.

In the subgroup analysis of participants who had been diagnosed for at least 12 months, the TIR (WMD 9.88%, 95% CI 6.72-13.04%, p < 0.01, I^2^ = 0%) (Supplementary Figure S4) and number of patients who reached HbA1c level < 7% (RR 3.74, 95% CI 1.89-7.39, p < 0.01, I^2^ = 0%) were greater in the HCL group. This group also had greater reduction in glucose level (WMD -15.10 mg/dL, 95% CI -22.32 to -7.88 mg/dL, p < 0.01, I^2^ = 0%) and HbA1c level (WMD -0.50%, 95% CI -0.69 to -0.31, p < 0.01, I^2^ = 0%) compared with the control group.

**Figure S5:**
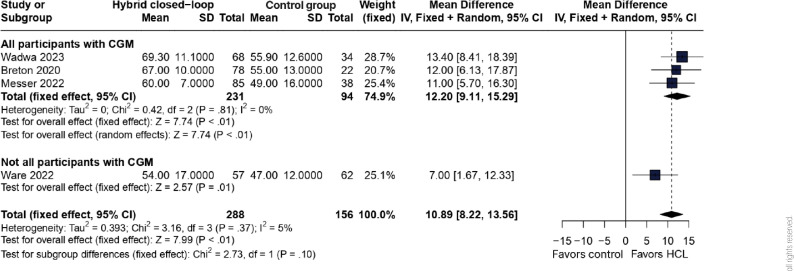
Forest plot of the time in range (TIR) analyzing participants with continuous glucose monitoring (CGM).

In the subgroup analysis including only control therapy with CGM, the TIR (WMD 12.20%, 95% CI 9.11-15.29%, p < 0.01, I^2^ = 0%) (Supplementary Figure S5) and number of patients who reached HbA1c level < 7% (RR 1.93, 95% CI 1.11-3.34, p = 0.02, I^2^ = 16%) were greater in the HCL group. This group also had greater reduction in glucose level (WMD -17.92 mg/dL, 95% CI -23.89 to -11.95 mg/dL, p < 0.01, I^2^ = 0%) and HbA1c level (WMD -0.50%, 95% CI -0.72 to -0.27%, p < 0.01, I^2^ = 0%) compared with the control group with CGM.

### Trial sequential analysis

**Figure S6:**
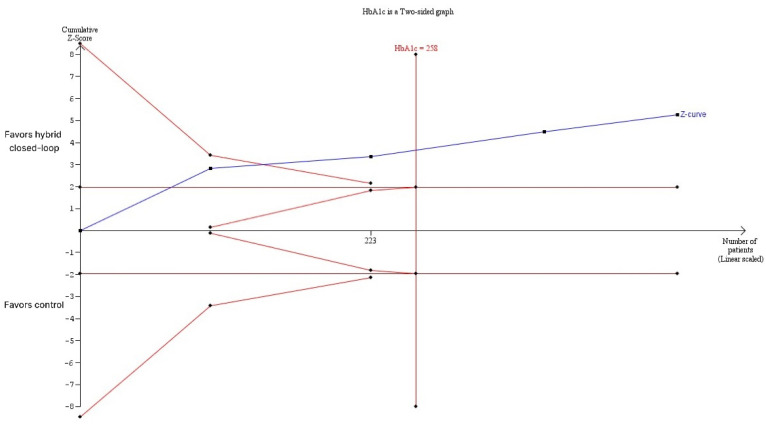
Trial sequential analysis for glycated hemoglobin (HbA1c) level.

**Figure S7:**
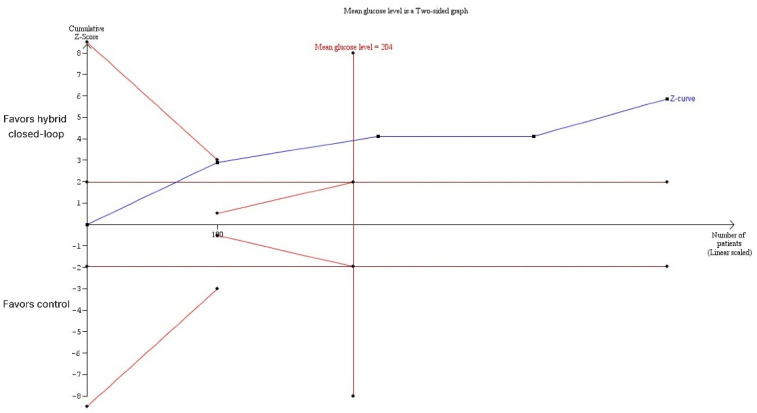
Trial sequential analysis for mean glucose level.

**Figure S8:**
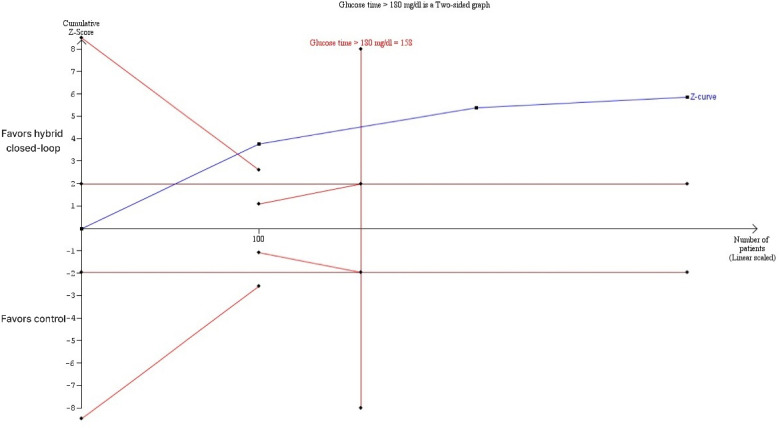
Trial sequential analysis for glucose time > 180 mg/dL.

**Figure S9:**
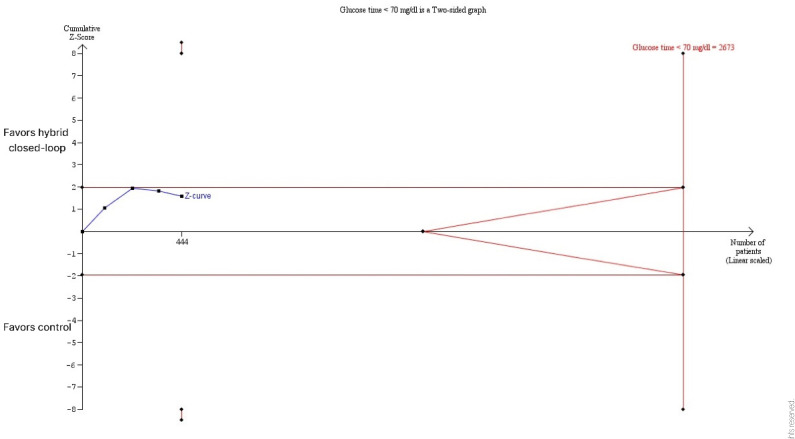
Trial sequential analysis for glucose time < 70 mg/dL.

**Figure S10:**
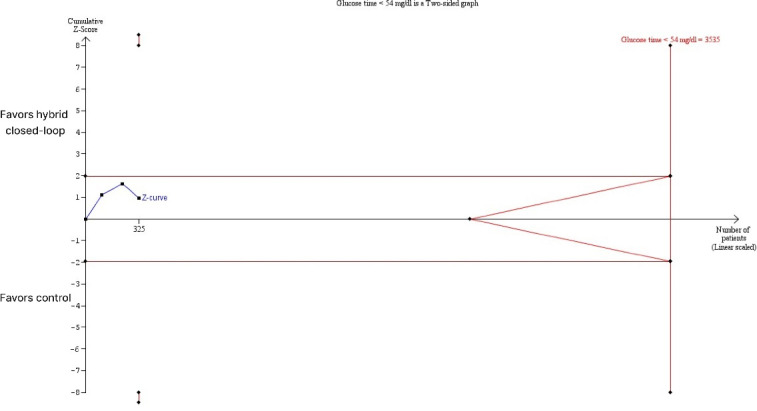
Trial sequential analysis for glucose time < 54 mg/dL.

**Figure S11:**
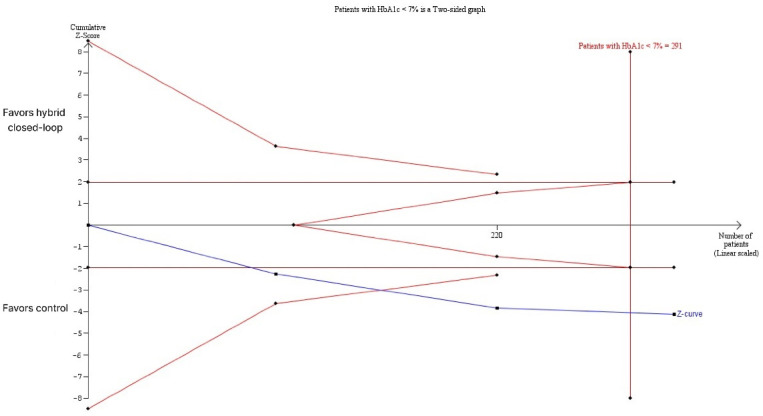
Trial sequential analysis of participants with glycated hemoglobin (HbA1c) < 7%.

The TSA showed firm evidence supporting the benefit of the HCL system over the control therapies regarding the outcomes of TIR, glucose time > 180 mg/dL, mean HbA1c level, mean glucose level, and number of patients with HbA1c level < 7%. However, for the outcomes of glucose time < 70 mg/dL and glucose time < 54 mg/dL, the analysis revealed a lack of firm evidence. The TIR results are shown in Figure 2. Further analysis of other outcomes is presented in Supplementary Figures S6, S7, S8, S9, S10, and S11.

### Sensitivity analysis

**Figure S12:**
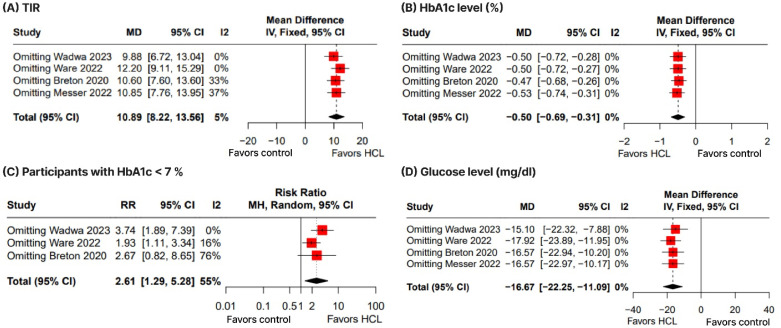
Leave-one-out sensitivity analyses. (**A**) Time in range (TIR). (**B**) Glycated hemoglobin (HbA1c) level (%). (**C**) Participants with HbA1c < 7%. (**D**) Glucose level (mg/dL).

**Figure S13:**
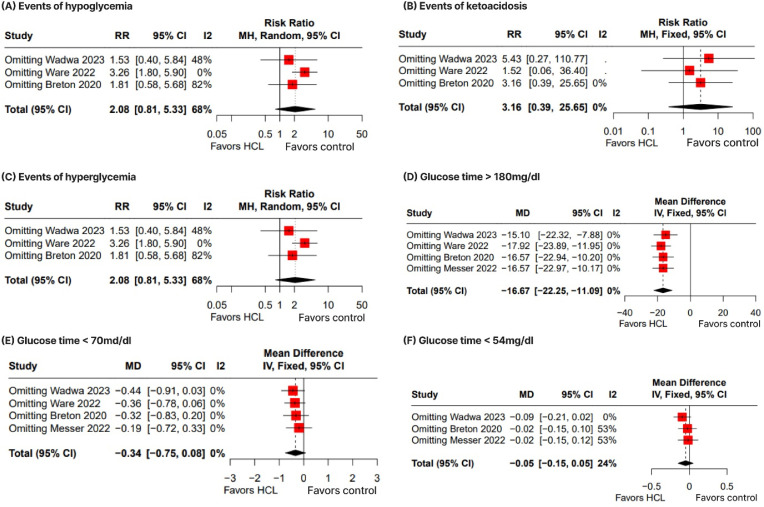
Leave-one-out sensitivity analyses. (**A**) Events of hypoglycemia. (**B**) Events of ketoacidosis. (**C**) Events of hyperglycemia. (**D**) Glucose time > 180 mg/dL. (**E**) Glucose time < 70 mg/dL. (**F**) Glucose time < 5 4 mg/dL.

The leave-one-out sensitivity analyses of all outcomes did not change the results. No significant changes in the statistical significance of pooled effect sizes were observed. All analyses are presented in Supplementary Figures S12 and S13.

### Risk of bias and publication bias

**Figure S15:**
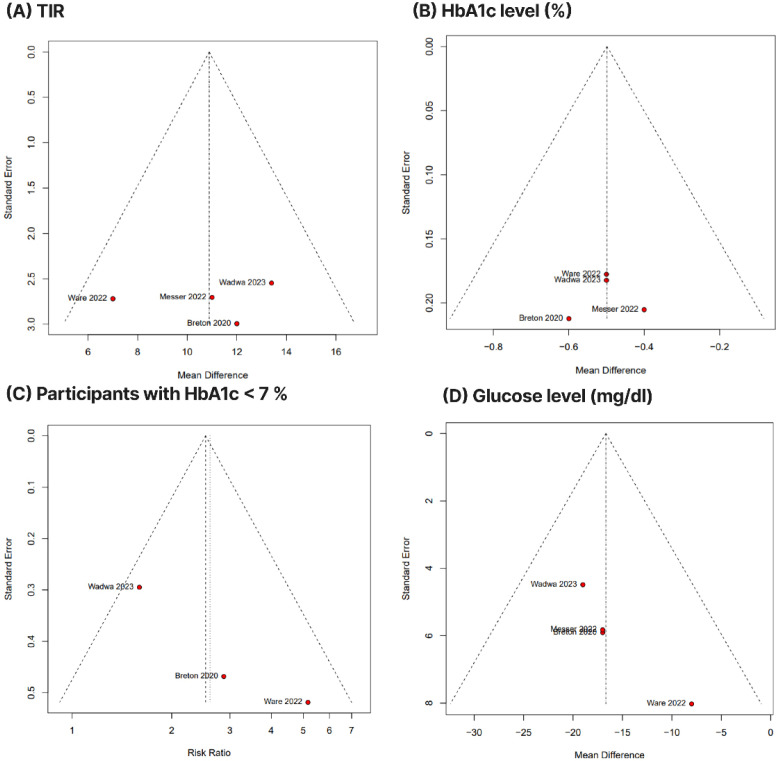
Funnel plot of the outcomes. (**A**) Time in range (TIR). (**B**) Glycated hemoglobin (HbA1c) level (%). (**C**) Participants with HbA1c < 7%. (**D**) Glucose level (mg/dL).

**Figure S16:**
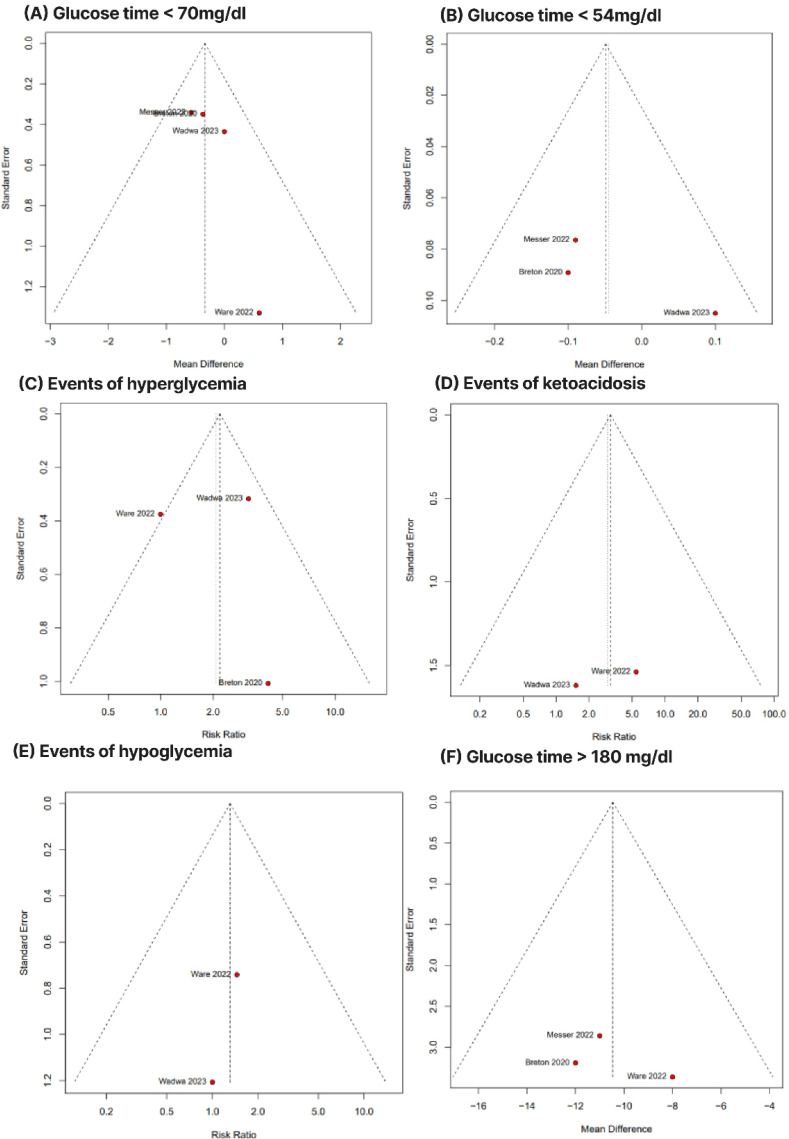
Funnel plot of the outcomes. (**A**) Glucose time < 70 mg/dL. (**B**) Glucose time < 54 mg/dL. (**C**) Events of hyperglycemia. (**D**) Events of ketoacidosis. (**E**) Events of hypoglycemia. (**F**) Glucose time > 180 mg/dL.

Appraisals of individual RCTs using the RoB 2 tool are shown in Supplementary Figure S17. Overall, the risk of bias in the RCTs was rated low in all five domains. An analysis of the funnel plots indicated possible publication bias due to studies lying outside the control lines for the outcome of hyperglycemia events. All the other outcomes had a low possibility of publication bias. The funnel plots of the outcomes are presented in Supplementary Figures S15 and S16.

According to the GRADE assessment, the following seven outcomes evaluated in the present study had quality of evidence rated low: TIR, glucose time < 70 mg/dL, glucose time < 54 mg/dL, glucose time > 180 mg/dL, HbA1c (%), glucose level (mg/dL), and number of patients with HbA1c < 7%. The low rating was mainly due to different pump models and algorithms in the HCL group. The quality assessment is detailed in the Supplementary Table.

## DISCUSSION

In this meta-analysis and TSA of four RCTs including over 500 children and adolescents with T1D, in which we compared the HCL system versus control therapies, the key findings were as follows: (A) the time spent in the target range was 10.89% longer (*i.e.*, approximately 2.6 hours/day) in children and adolescents using the HCL system, (B) the mean glucose level and (C) percentage of time during which glucose level was >180 mg/dL was decreased with the HCL system, and (D) the mean HbA1c level was lower in the HCL group, consequently, (E) more patients in this group reached an HbA1c level <7% when compared with the control group. Additionally, no significant differences were observed regarding the number of events of hyperglycemia, severe hypoglycemia, or ketoacidosis.

Achieving target glucose and HbA1c levels within TIR reduces the risk of acute and chronic complications and minimizes the detrimental effects of hypoglycemia and hyperglycemia on brain development, cognitive function, mood, and quality of life (19,35–37). To the best of our knowledge, this is the first meta-analysis and TSA comparing the HCL system with other control therapies exclusively in children and adolescents with T1D. Other meta-analyses have only evaluated the efficacy and safety of the HCL system in adults (individuals older than 18 years) and adolescents. These studies did not present distinct outcomes for each population or perform separate analyses for the two groups (20). The most recent meta-analysis on this topic encompassed a combination of observational studies and RCTs but did not incorporate the findings from the three latest published RCTs (18,19). Beck and cols. conducted a thorough meta-analysis using individual patient data but did not perform a systematic review, which may have introduced some bias in the generalization of results (38). We focused on children and adolescents aged 2 to 18 years with a diagnosis of T1D. Although this age group presents unique challenges in achieving glycemic goals during the dynamic phase of growth, it has often been underrepresented in previous studies.

Although a lower HbA1c level has been historically considered a risk factor for severe hypoglycemia, this association is no longer observed with intensive management supported by diabetes technology (39,40). In the present meta-analysis of RCTs including children with mean ages ranging from 3.84 to 13.1 years, the absence of difference between HCL and control therapies regarding glucose time < 70 mg/dL and glucose time < 54 mg/dL is certainly reassuring. Although there was no difference in hypoglycemia time, the use of an HCL system was related to a decrease in HbA1c level and a greater number of patients who reached the HbA1c target level of < 7%. These findings are in line with previous systematic reviews that demonstrated the safety of different HCL devices without increased risk of hypoglycemia (21,22).

Although our pooled analysis indicated no significant differences between groups regarding cases of hyperglycemia, the percentage of time in which glucose level was > 180 mg/dL was lower in the HCL group. Therefore, despite reducing the duration of hyperglycemia, the HCL system is still associated with a relevant number of acute hyperglycemic events, probably due to infusion set failures. This observation could be related to the lack of significance regarding the number of hyperglycemic events in our analysis (41). Of note, the number of cases of hyperglycemia with HCL compared with control therapies in three of the included RCTs were, respectively, 51 and 8 (Wadwa and cols., 2023) (5), 14 and 1 (Breton and cols., 2020) (9), and 11 and 12 (Ware and cols., 2022) (34). However, two of these trials had a significant decrease in time during which glucose level was > 180 mg/dL in the HCL group, showing that the cases of hyperglycemia are potentially due to specific insulin pump management issues (9,34). In the RCT by Messer and cols. (2022), data on adverse events were not available for children without previous use of the HCL system, precluding the use of this trial for assessment of these outcomes (33).

In TSA, firm evidence is reached when the number of patients in the study exceeds the required number for a meta-analysis to accept or reject the intervention, and when the z-curves cross the conventional boundaries (z = 1.96) value. Also, firm evidence can be established if the z-curve crosses the calculated trial sequential monitoring boundaries (TSMBs) before reaching the number of patients required to accept or reject the intervention. When the number of patients does not reach the required threshold and the z-curve crosses the conventional boundaries but not the TSMBs, the result may be spurious due to possible random error resulting from repetitive testing (32). All the outcomes that demonstrated a significant difference in favor of the HCL group in our meta-analysis showed firm evidence in the TSA. For the TIR, the percentage of time during which glucose level was > 180 mg/dL, mean HbA1c level, mean glucose level, and number of patients with HbA1c < 7%, the z-curves crossed both TSMB and the required number of patients to accept the intervention. This suggests that the current evidence is sufficient, and further studies are unlikely to change the conclusion of the similarity in safety profiles between HCL systems and control therapies (32).

Our study has limitations that must be acknowledged. First, the RCTs that enrolled children with T1D had small sample sizes and short follow-up periods, which potentially underpowered our analysis and amplified the chances of a type I error. Second, the inclusion of heterogeneous age groups in the meta-analysis was due to a small number of studies available, which made it impossible to assess the effectiveness of a model and potential usability issues according to patients’ age. Also, the small number of trials included may not provide a meaningful interpretation of the publication bias and funnel plot. Third, the studies used different pump models and algorithms within the HCL group, lacking representativeness of the open-source automated insulin delivery system (42,43). Fourth, crossover studies were excluded due to concerns regarding potential bias in the trial design. However, this decision may introduce a limitation, as the majority of the trials conducted with HCL systems often employed a crossover design. Fifth, the control therapies varied and included from conventional pumps with or without CGM to sensor-augmented pumps, low-glucose suspend, predictive low-glucose suspend, and multiple daily injections with CGM use. To minimize the potential influence of certain limitations, we performed subgroup analyses to explore the hypotheses of whether different pump models, the role of CGM, and a time greater than 12 months since diagnosis were associated with different outcomes. No significant differences were observed among the subgroups. Finally, the absence of data related to costs precluded the assessment of this barrier, which is one of the significant challenges associated with access to diabetes technology (44,45).

We may infer from the results of the present study that the HCL system represents a paradigm shift in the lives of individuals with T1D and their families (41,46,47). This therapy needs to be adapted to comprehend the entire childhood period, considering the unique needs of toddlers and preschoolers (17,48). The greatest challenge in the years ahead is to ensure that these technologies reach every child and adolescent across the world (49).

In conclusion, in this systematic review and meta-analysis, the HCL system was associated with improved TIR and HbA1c control in children and adolescents with T1D compared with control therapies, with a similar profile of side effects. These findings support the efficacy of HCL in the treatment of T1D in this population. The TSA indicated that we presented firm evidence and that new studies are unlikely to change the results.

## Figures and Tables

**Figure S14:**
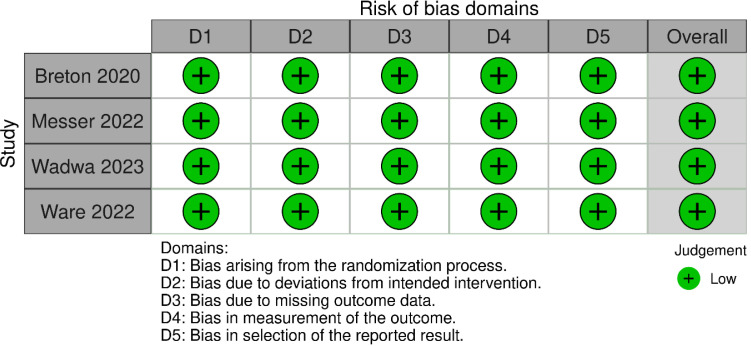
Critical appraisal of randomized controlled trials according to the Cochrane Collaboration’s tool for assessing risk of bias in randomized trials.

## SUPPLEMENTARY TABLE

**Table:** Hybrid closed-loop (HCL) system compared with control therapies in children and adolescents with type 1 diabetes Bibliography: Ferreira et al.

Certainty assessment							Summary of findings				
Participants (studies) Follow-up	Risk of bias	Inconsistency	Indirectness	Imprecision	Publication bias	Overall certainty of evidence	Study event rates (%)		Relative effect (95% CI)	Anticipated absolute effects	
							With control group	With HCL		Risk with control group	Risk difference with HCL
**Glucose time 70-180 mg/dL**											
444 (4 RCTs )	not serious	very serious^a^	not serious	not serious	none	⨁⨁◯◯ Low	156	288	-		MD **10.89 higher** (8.22 higher to 13.56 higher)
**Glucose time <70 mg/dL**											
444 (4 RCTs)	not serious	very serious^a^	not serious	not serious	none	⨁⨁◯◯ Low	156	288	-		**MD 0.34 lower** (0.75 lower to 0.08 higher)
**Glucose time <54 mg/dL**											
325 (3 RCTs)	not serious	very serious^a^	not serious	not serious	none	⨁⨁◯◯ Low	94	231	-		**MD 0.05 lower** (0.15 lower to 0.05 higher)
**Glucose time >180 mg/dL**											
342 (3 RCTs)	not serious	very serious^a^	not serious	not serious	none	⨁⨁◯◯ Low	122	220	-		**MD 10.46 lower** (13.99 lower to 6.93 lower)
**HbA1c (%)**											
458 (4 RCTs)	not serious	very serious^a^	not serious	not serious	none	⨁⨁◯◯ Low	162	296	-		**MD 0.5 lower** (0.69 lower to 0.31 lower)
**Glucose level (mg/dL)**											
444 (4 RCTs)	not serious	very serious^a^	not serious	not serious	none	⨁⨁◯◯ Low	156	288	-		**MD 16.67 lower** (22.25 lower to 11.09 lower)
**HbA1c <7%**											
315 (3 RCTs)	not serious	very serious^b^	not serious	not serious	none	⨁⨁◯◯ Low	18/118 (15.3%)	88/197 (44.7%)	**RR 2.61** (1.29 to 5.28)	153 per 1.000	**246 more per 1.000** (from 44 more to 653 more)

Explanations

**CI:** confidence interval; **MD:** mean difference; **RR:** risk ratio

a Different pump models and algorithms for the hybrid clos ed-loop group; different control therapy.

b High heterogeneity; different pump models and algorithms for the hybrid clos ed-loop group.
